# Qualitative Insights Into Non-attendance for Scheduled Radiology Appointments at a Specialist Hospital in Saudi Arabia

**DOI:** 10.7759/cureus.79700

**Published:** 2025-02-26

**Authors:** Majed Alturbag, Mary Mooney, Gabrielle McKee, Margarita Corry

**Affiliations:** 1 School of Nursing and Midwifery, Trinity College Dublin, Dublin, IRL

**Keywords:** patient non-attendance, qualitative research, radiology appointments, radiology & imaging, thematic analysis

## Abstract

Background: Patient non-attendance for radiology appointments is an international problem with significant implications for healthcare recipients and healthcare efficiency. Non-attendance impacts patient health, waiting lists, and other hospital departments while increasing staff stress, anxiety, and fatigue. Understanding the reasons behind patient non-attendance is crucial for developing effective strategies to help improve attendance rates.

Objective: This study explored the reasons for patient non-attendance for scheduled radiology appointments and identified potential strategies to enhance attendance at a specialist hospital in Saudi Arabia.

Methods: Using semi-structured interviews, nine men and eight women who were purposively sampled and had missed scheduled radiology appointments at the research site were interviewed. Thematic analysis was employed to identify the key themes represented by the data.

Findings: This qualitative study revealed the multifaceted nature of patient non-attendance for scheduled radiology appointments at the specialist hospital in Saudi Arabia. Five themes underlying non-attendance were identified. First, scheduling conflicts were a significant barrier. Second, a lack of adequate knowledge about health conditions was evident. Third, physician-patient miscommunication was a critical issue. Fourth, transportation difficulties, especially for those living far from the hospital or without personal transportation, were a key factor in non-attendance. Finally, personal reasons, such as fear of medical procedures and the patient’s health status, also contributed. The study identified two main areas for improvement: implementing an effective appointment reminder system and enhancing the radiology department by extending hours and addressing non-attendance more effectively. These strategies underscore the need for a patient-centered approach to reduce barriers to attendance.

Conclusion: The findings suggest that patient non-attendance is multifactorial, involving personal and hospital-specific reasons. Strategies to improve attendance should thus be multifaceted, including better scheduling systems, enhanced patient education and communication, and reminder systems. These insights can inform targeted interventions to reduce non-attendance rates, ultimately improving healthcare delivery and resource utilization.

## Introduction

Non-attendance impacts the operational efficiency of hospitals, leading to longer waiting times for patients and often exacerbating health issues due to delayed diagnosis or treatment. Understanding the reasons for non-attendance can help mitigate these challenges, ultimately enhancing patient care and resource management.

Drawing on a range of international studies, this study found many reasons for non-attendance, which can be grouped into patient-centered and hospital-specific categories.

Patient-centered reasons for non-attendance have been widely documented across various countries, including the United Kingdom, Spain, Ireland, Germany, Nigeria, Argentina, the United States, Australia, and the Sultanate of Oman [1-11]. Among them, forgetfulness was widely cited as a primary cause of non-attendance. Poll et al. (2017) and Alhamad (2013) highlighted the multifaceted nature of forgetfulness, indicating that reasons such as late appointment notifications could contribute to the problem [12,13]. Moreover, significant barriers to attendance include conflicts between patients’ personal work commitments or other schedules and hospital appointment times. Another patient-centered factor of note is a dependence on third parties for transportation or accompaniment, which may be the situation for some older patients, while in the case of females, where sole travel is forbidden, a third party or chaperone is required [1,14-16].

Hospital-specific reasons also contribute to appointment non-attendance. Extended waiting times, challenges in managing appointments, and the nature of the patient-physician relationship were all identified from the literature as key contributors to non-attendance. Studies have indicated that prolonged waiting times when booking an appointment and extended clinic waiting times increased non-attendance rates [8,17,18]. The lack of reminders and communication breakdowns in appointment management were further highlighted as significant barriers to patient attendance at hospital appointments [11,16,19-21]. Moreover, the dynamics of the doctor-patient relationship, including the effect of trust and clear communication, can influence patient attendance [18,22].

In radiology departments, the efficient scheduling and utilization of sophisticated imaging equipment and specialized personnel are paramount. However, few studies heretofore have focused on radiology departments, an area we aimed to address. This study comprehensively explored the reasons underlying patient non-attendance at radiology appointments, considering both patient-centric and hospital-specific reasons. Moreover, this study engaged directly with patients to determine potential solutions for improving attendance rates. The aim of this study was to explore the subjective experiences and perceptions of these individuals to gain an understanding and insight into the reasons for hospital non-attendance. This study was driven by the following question: What are patients’ reasons for non-attendance at appointments in the radiology department of the specialist hospital?

## Materials and methods

Research design and context

The study was conducted at King Fahad Specialist Hospital, Saudi Arabia. A phenomenological research design approach was utilized to explore and understand the lived experiences of participants concerning missed appointments. Phenomenology seeks to uncover the essence of individuals' experiences with a specific phenomenon. This approach emphasizes exploring participants' subjective perspectives and meanings. By focusing on the personal narratives of participants who had experienced the phenomenon of missed appointments, the study aimed to identify their reasons and thought processes. The researcher engaged in bracketing to set aside personal biases, ensuring an unbiased interpretation of participants’ experiences and allowing for a comprehensive understanding of the phenomenon.

Study participants

A qualitative descriptive research design was utilized. We explored patients’ reasons for missing their scheduled appointments in the radiology department and gained an in-depth understanding of the issue from patients’ perspectives. Additionally, we interacted with patients to devise possible strategies for enhancing attendance rates. To identify suitable participants, this investigation employed purposive sampling means which included selecting participants who met the eligibility criteria and had firsthand experience with the phenomenon of interest, i.e. missed scheduled appointments. Eligibility for participation was determined based on individuals being at least 18 years old, having a recent history of missing appointments (within the past seven days), and possessing the legal capacity to provide informed consent. Ethical approval was granted in full by the hospital and university (20180419). The study was conducted over a duration of eight months, from June 2021 to February 2022.

Recruitment process

The recruitment strategy involved the appointment of a gatekeeper, who in this study also acted as a research assistant. The agreed-upon procedure involved the assistant compiling a weekly roster of study-eligible patients who had failed to attend their scheduled appointments in the department. The assistant telephoned 287 potential participants. Of these, 46 individuals expressed interest in the study. Of these consenting individuals, 21 contacted the researcher for further information and agreed to participate in the study. Interviews were scheduled with these participants, considering their preferred time, date, and location. Of the 21 individuals who initially agreed to participate, three failed to attend the scheduled interviews and did not respond to subsequent follow-up calls. Additionally, one participant withdrew from the interview process. Consequently, the final sample size was 17 participants. The demographic characteristics of these participants are detailed in Table 1.

**Table 1:** Participant characteristics

Parent ID number	Gender	Nationality	Age	Marital Status	Educational Qualification
P1	Male	Saudi	61 and above	Married	Primary school
P2	Female	Saudi	18-28	Single	Bachelor’s degree
P3	Female	Saudi	29-39	Single	Bachelor’s degree
P4	Male	Saudi	51-61	Married	Master’s degree
P5	Male	Saudi	29-39	Married	Bachelor’s degree
P6	Female	Saudi	29-39	Married	Master’s degree
P7	Female	Saudi	40-50	Widowed	Bachelor’s degree
P8	Female	Saudi	29-39	Single	Bachelor’s degree
P9	Male	Saudi	18-28	Married	Bachelor’s degree
P10	Male	Saudi	18-28	Single	Diploma
P11	Female	Saudi	18-28	Divorced	Bachelor’s degree
P12	Male	Saudi	29-39	Single	Diploma
P13	Male	Egyptian	29-39	Married	Bachelor’s degree
P14	Male	Saudi	18-28	Single	Attended university
P15	Male	Saudi	18-28	Single	In the third year at university
P16	Female	Saudi	18-28	Single	High school
P17	Female	Saudi	40-50	Married	Bachelor’s degree

Data collection

We used semi-structured interviews to guide the data collection. The interview questions were informed by the literature and were specifically crafted to elicit in-depth responses pertinent to the study's aim.

Interview procedure

To establish a comfortable environment for the interviews, a protocol was followed to put participants at ease and build rapport. Upon arrival, each participant was engaged in light conversation, and refreshments were offered. Before the interview, the researcher described the purpose of the study and sought informed consent. The researcher conducting the interviews was skilled in handling the sensitive aspects of participant interaction, such as confidentiality and cultural and ethical considerations.

The questions were open-ended. The interviews varied in length, typically lasting 25-40 minutes. With the consent of the interviewees, these sessions were audio-recorded, and in addition to this, written notes were taken. Sociodemographic data were collected directly from the participants (Table 1).

In total, 17 interviews were conducted. To safeguard the privacy of the participants, a systematic coding approach was employed to label interview records from P1 to P17. Objectivity was maintained as far as was reasonable throughout the interview process, with respect for the opinions and perspectives shown through body language, tone, and expressions of interest.

Data analysis approach

**Figure 1:**
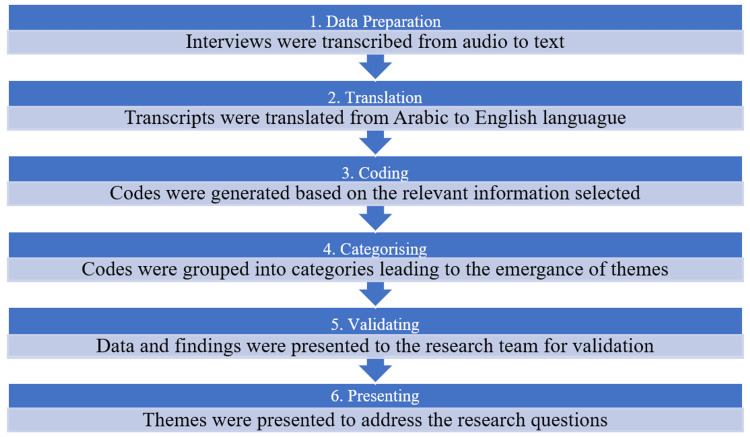
The process of thematic analysis in the current research (prepared by the researcher)

Thematic analysis for this study was employed (Figure 1). Thematic analysis is particularly effective in extracting meaningful patterns and themes from qualitative data, allowing for a deeper understanding of the participants’ experiences and perspectives. This approach is instrumental in uncovering truths, augmenting existing knowledge, and unveiling insights relevant to the research question [23].

The thematic analysis in this study was methodically conducted through a structured six-step process, as illustrated in Figure 1. Initially, the audio recordings of the interviews were transcribed verbatim. The original transcriptions were in Arabic, the spoken language of the participants. Subsequently, these Arabic transcripts were translated into English. To ensure accuracy and fidelity, a professional translator was enlisted. The translated content was then rigorously assessed for consistency and correctness. The research team, who did not speak Arabic, revisited the translated transcripts, revising, extracting, and refining codes to reflect the data or eliminating those not addressing the research question. At the end of this process, the researchers presented sixty-one codes relating to the research question. Twenty-nine codes were selected for intervention strategies. In categorization, researchers put codes that were similar in nature into the same cluster. The sixty-one codes associated with reasons for missed appointments were grouped into ten clusters. The researchers further examined the categories and grouped them into five clusters. The twenty-nine codes generated under the patients’ suggested intervention strategies for the missed appointments were grouped into two clusters.

## Results

Emergent themes from the interviews

Analysis of the interview data yielded significant insights into the reasons behind non-attendance. Five primary themes emerged, summarizing the various conditions contributing to non-attendance. These were scheduling problems (i.e., challenges in aligning appointments with personal schedules), inadequate knowledge and a misunderstanding of one’s health issues, physician-patient miscommunication, travel problems, and miscellaneous reasons. In addition to these themes, the study also revealed two potential solutions to reduce non-attendance: appointment reminders, such as text messages or phone calls, and departmental improvements to facilitate appointment attendance.

Themes underlying non-attendance

**Figure 2:**
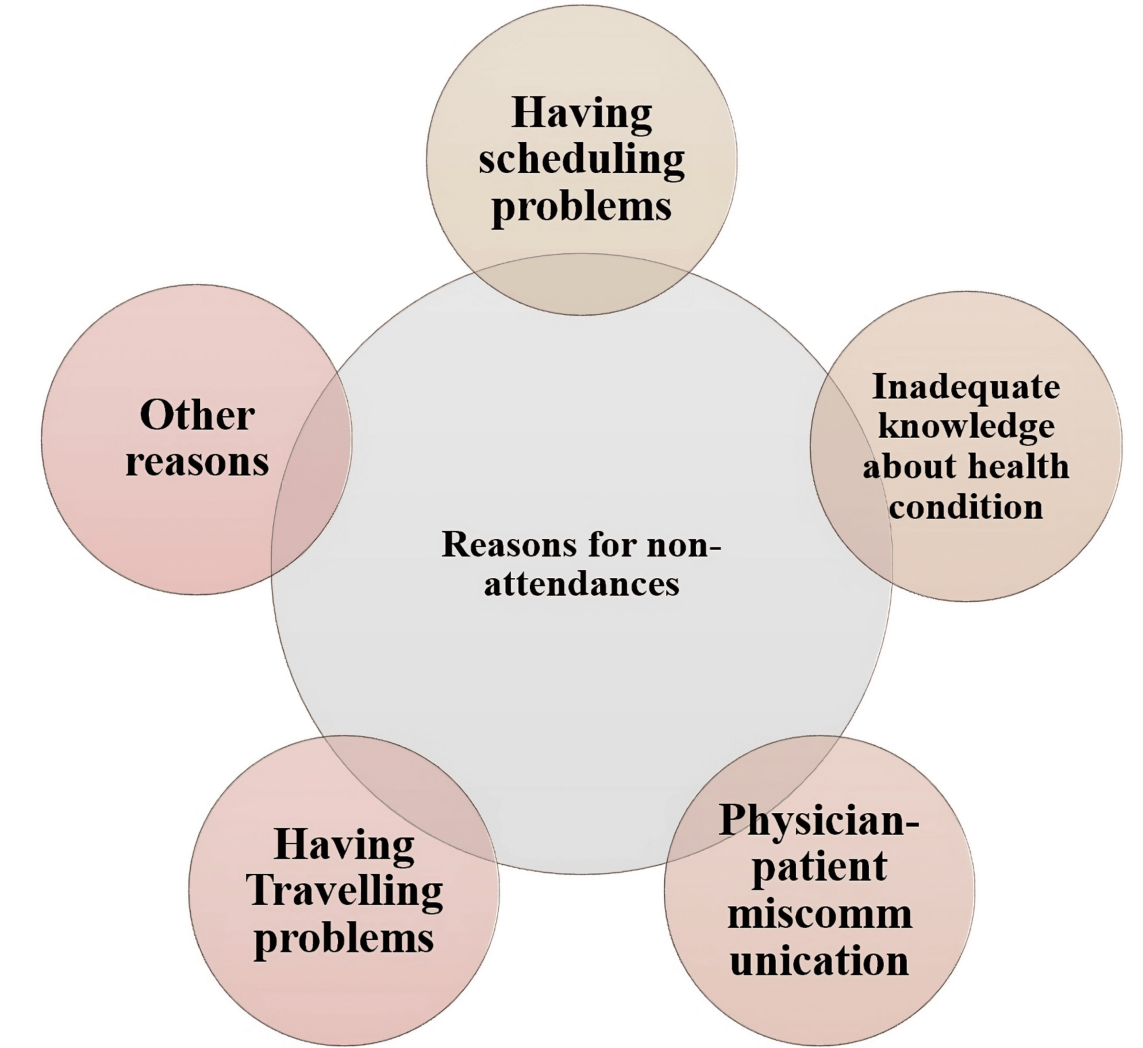
Patient's reasons for non-attendance

The reasons for appointment non-attendance were categorized into five distinct themes, offering a comprehensive understanding of the underlying causes (Figure 2).

Scheduling Problems

This theme reflects the combination of personal forgetfulness and systemic problems in the appointment scheduling process, underlining the need for improved scheduling practices and reminder systems to aid patients in managing their hospital appointments effectively. Two predominant concerns were related to scheduling conflicts and dissatisfaction with the long waiting periods between scheduling an appointment and the appointment date given. Additionally, it was said that the appointment management system did not provide suitable and convenient appointment times, all of which were said to contribute to non-attendance.

Forgetfulness, waiting, and lack of reminders: A notable reason for non-attendance was participants forgetting their appointments (Table 2).

**Table 2:** Themes for the reasons for non-attendance and their characteristics

Theme	Case count (i.e., the number of participants associated with the theme)	Evidence
Having scheduling problems	30	“You have a big problem, which is that there are no options when it comes to appointments. For, in the morning, I work in the court, and it is difficult for me to leave work to do tests.” (P10) “As for my own reasons, it is because the appointment was scheduled three or four months ago, and I simply forgot about it and also the hospital did not remind me.” (P8)
Inadequate knowledge of health conditions	3	“My health condition is stable; therefore, no serious problems will affect my health since it is a normal thing and happens to some girls. In addition, there are no complications, and this is based on what I got from some of my friends since I asked them about it. Also, some of them talked about some medication that can help manage and regulate the menstrual cycle so if I wanted to try them, they will aid me.” (P16)
Physician-patient miscommunication	3	“He did not explain anything to me at all, and I do not know why I was referred.” (P1)
Having travelling problems	4	“Another obstacle is driving, since I am a woman who doesn’t drive, and I have to try to coordinate with my husband, who is employed.” (P6)
Other reasons	5	“I immediately decided on the same day that I attended the hospital, to head to another private hospital and get the scan done instead of waiting for three days. So, I did the scan the same day.” (P6)

This issue was particularly pronounced when there was a significant gap between the date of scheduling, the appointment, and the actual appointment date. Seven participants noted that they had forgotten their appointment because of this extended interval. As one participant stated,

"As for my own reasons, it is because the appointment was scheduled three or four months ago, and I simply forgot about it and also the hospital did not remind me." (P8)

Confusion among some about the exact appointment date resulted in instances of non-attendance. For example, one participant shared their experience of attending on the wrong day due to confusion:

"I was completely confused about the day of the appointment. My appointment was actually on Wednesday but, unfortunately, I attended the hospital at half past eight am on the Thursday. Then, when I gave the employee the appointment slip, she informed me that my appointment was yesterday, and I did not realise that until the moment she told me." (P15)

Another significant reason for non-attendance was the prolonged wait for radiology services, particularly MRI procedures. This lengthy waiting period was a source of dissatisfaction among patients and was directly linked to their absence from scheduled appointments. Three participants cited the long waiting time as the primary reason for missing their appointment, and of note, all had appointments booked for MRIs. One participant shared a sentiment, highlighting the challenge of remembering an appointment scheduled far in the future:

"Personally, I find that the main reason was forgetting about it, I mean forgetting about the appointment. When you are given an appointment in five months’ time, do you think you will remember it?" (P5)

A further issue was the lack of appointment reminders from the hospital. Patients who were not familiar with the system generally expected to receive a reminder as their appointment date approached, and the absence of such reminders led to non-attendance. Three participants mentioned this issue, with one stating,

"I simply forgot about it and also the hospital did not remind me. However, this does not mean it is the hospital’s fault... no, because it is my appointment, and it is my fault but I am only describing exactly what happened." (P8)

Juggling appointments and life: Participants expressed concerns about the hospital’s appointment management system, including failure to answer calls and no clear information on how to reschedule or cancel appointments. One participant described their frustration with the communication process,

"Explain to me how I can do that when you essentially do not answer calls. The reality is different. I remember that I kept calling, and the only person who answered my calls was the operator, who transferred me (to the radiology department) where I got no response." (P9)

Another participant noted the lack of information on rescheduling:

"I realised it was late, and I didn’t know how I could reschedule it. I did not find a message to help me, such as, if you cannot attend, call this number... you know what I mean." (P10)

Additionally, the absence of a clear policy for handling non-attendance and a lack of consequences for non-attendance were mentioned by participants who noted,

"There must be a policy, and it should be followed. The absence of any policy means the problem will continue. Suppose the patient booked more than one appointment and did not attend; the problem will continue. Unfortunately, this is unclear for patients because there is no general framework for them to understand their rights and duties." (*P4)*

Poor timing: This theme reflects participants’ difficulties in aligning their appointments with personal and professional commitments. The challenges of finding a suitable time for appointments were compounded by work responsibilities, family obligations, and, in this context, the availability of family members for accompaniment, particularly for female participants. One participant described the struggle of balancing work commitments with department appointments,

"The issue is when your appointment falls within your working hours, you try to combine attending the appointment and committing to it and being careful not to abandon your official working hours. But ultimately, you face a difficulty in doing so, for example, being under pressure at work so your direct manager does not allow you to leave work and go to the hospital."(P9)

A female participant highlighted her reliance on family members for transportation and how conflicting schedules could lead to non-attendance:

"My brother was going to attend the appointment with me because he is the one who has a car and drives me. However, it just so happened that he had a job interview in Riyadh that day, so he had to travel to attend that interview." (P2)

Inadequate Knowledge About Health Conditions

Participants also indicated that a lack of understanding or awareness of their health condition influenced their decision to miss appointments. This theme encapsulates both the low perceived need to attend appointments and insufficient knowledge about the seriousness of their condition. One participant expressed skepticism about the value of attending their radiology appointment, doubting the necessity and efficacy of the visit:

"In my case, there is no need. It is a complete waste of time, and there is nothing that will be identified regarding my condition. Also, if it was significant or added anything, I would have benefited from the last time that I visited the doctor." (P16)

The impact of not perceiving one’s health condition as serious or disruptive was also noted:

"It’s been eight months since I first noticed that feeling, and it hasn’t affected my daily life." (P10)

Additionally, the availability of alternative treatments and advice from others was mentioned by a participant who was considering traditional methods over scheduled medical appointments:

"I used traditional medicine to treat my problem. One of my friends advised me to put my knee in the sand and cover it and I have seen a great response in my knee from that, but I did not continue this traditional medicine method." (P1)

Physician-Patient Miscommunication

This theme encompasses aspects related to the quality of interaction and communication between physicians and patients, as well as issues of trust and understanding. Participants expressed concerns about not being fully informed or involved in discussions regarding their medical tests and treatments. One participant illustrated this lack of clarity in communication:

"You know, they did electroencephalography again and clinical investigation and did not focus on my complaint, then you attend the clinic and see your consultant … who just says, keep taking the medication you are on, and you need to go to the X-ray department. And I’m saying to him, I can’t understand it and my problem needs treating because I always feel dizzy and have a headache." (P17)

Another participant highlighted language barriers in communicating their medical condition:

"The doctor at the primary care facility did not write in Arabic about my condition." (P1)

There were also concerns about the perceived inexperience or hesitance of physicians, which affected patients’ confidence in their care:

"I think he was somewhat hesitant. He is not an experienced doctor, because he is a young doctor. I also don’t think he has the required experience." (P10)

Travel Problems

Travel and transportation to the hospital were significant barriers for some participants, especially for those living at a considerable distance or without personal transportation. One participant explained the difficulty in finding someone to provide transportation:

"I will be clear and honest with you. The issue is not that I forgot my appointment, but it lies in the fact that I couldn’t find anyone to drive me to the appointment like my brother who I depend on for transportation." (P2)

A female participant described the challenge of coordinating transportation with a working family member:

"Another obstacle is driving, since I am a woman who doesn’t drive, and I have to try to coordinate with my husband, who is employed." (P6)

Insufficient transportation facilities were highlighted as a problem not only for women but also for any patient without a personal means of transport, as another participant said,

"…the lack of transportation facilities makes travelling a bit complicated. Not just for a woman but also for a man if they don’t have their own car or someone to drive them." (P6)

Other Reasons

Several less frequently mentioned reasons for non-attendance were also identified. One participant chose an alternative option due to the urgency of their condition:

"I was complaining of constipation and contractions in my abdomen. But I went to another doctor who gave me medication, some laxatives and ointments. After that, I was not complaining of the problem anymore. Because of that, I believed that I no longer needed the appointment and also, since the doctor told me that there was no need for the scan that was required, I believed that I no longer needed the scan." (P11)

Health issues immediately preceding the appointment were a barrier for another participant:

"My appointment was scheduled for Sunday last week but the day before, on Saturday, I felt really ill, with tremendous exhaustion and a fever, and so I went to the ED, where I was in a critical condition. Additionally, I threw up every time I took my medication, indicating that my situation was unstable. At the ED, because the doctor was unsure whether the symptoms, I was experiencing were COVID-19 symptoms or were related to my illness or were caused by something else, I took a COVID swab test, which came back negative, but I continued to feel unwell until Sunday, the day of the examination appointment. As a result, I was unable to attend as I was bedridden and couldn’t move properly." (P3)

Fear and anxiety about the potential outcomes of medical appointments were also significant deterrents for some:

"It’s intimidating when you’re about to... When you go to a clinic or an appointment, you never know what they’re going to tell you. Um, it was just some negative ideas that were running through my brain about the matter." (P7)

Issues with medical insurance also played a role in non-attendance:

"I was determined to attend. However, this required the agreement of the insurance company, to which we had already applied for approval to cover the scan expenses, but, unfortunately, the insurance company did not agree to the scan request." (P13)

These themes address the diverse reasons underlying non-attendance, ranging from practical and logistical challenges to personal perceptions and fears. A comprehensive understanding of patient experiences is clearly needed to improve healthcare delivery.

Enhancing attendance through strategic interventions

**Figure 3:**
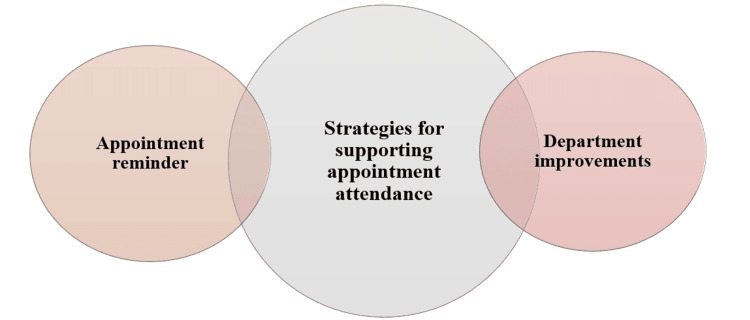
Strategies for supporting appointment attendance

The study also identified two key themes that emerged from participants’ suggestions on improving attendance at radiology department appointments. These themes, centered around appointment reminders and department improvements, reflect the patients’ perspectives on practical solutions to the issue of non-attendance (Figure 3).

Appointment Reminders

This theme revolved around participants’ preferences for reminders of their appointments. Most participants emphasized the need for an effective reminder system, suggesting various methods such as electronic reminders and phone calls. The convenience of text message reminders was highlighted by several participants. For instance, one participant appreciated this method, saying,

"My dentist uses text messages to remind me of my appointments. It would be cool if you could do something like that, you know? I just forget appointments if I made them a long time ago." (P8)

Given the widespread use of smartphones and social media, seven participants suggested leveraging these platforms for reminders. One participant expressed a preference for WhatsApp (Meta Platforms, Inc., Menlo Park, California, United States), noting,

"In my opinion, it’s to use WhatsApp as I’m constantly connected to the internet and rarely speak on the phone these days as everything is done via social media, whether it’s Snapchat, WhatsApp, or Instagram. Our lives have shifted dramatically." (P3)

Another participant echoed this sentiment, emphasizing the popularity of WhatsApp in Saudi Arabia:

"Everyone uses WhatsApp in Saudi Arabia because it is a safe and inexpensive medium. Most people have it, and it is more popular than any other available means of communication. Just by looking at whoever is around, from colleagues to friends, you will see that they all use WhatsApp." (P2)

An approach combining text messages with phone calls was also favored by some participants (Table 3).

**Table 3:** Themes for the strategies for supporting appointment attendance and their characteristics

Theme	Case count (i.e., the number of participants associated with the theme)	Evidence
Appointment reminder	17	"I prefer receiving a phone call rather than a recorded call because it gives me the space to listen and answer and to inquire or change the appointment if I want, compared to a recorded call, which is a machine that will not understand you nor give you what you want." (P7)
Improvements in the department	13	"It is tough for me to attend the appointment while I am at work in the morning, and I am unable to go since we are understaffed. Perhaps you might extend the working hours till the evening or even on weekends so that I can attend my appointments.” (P10)

As one stated,

"A text message combined with a phone call would be great because if I don’t answer the phone, there will be a text message for me." (P5)

The personalization of reminders was also seen as a beneficial strategy. One participant suggested,

"It’s nice to see the doctor’s name at the end of a reminder, whatever the reminder is for. I think that it would lead to higher levels of appointment attendance by patients as they will appreciate what they see as a considerate gesture by the doctor." (P17)

The value of a phone call lay not just in the reminder itself but also in the personal touch it provided. As a participant explained,

"Phone calls are superior because they serve two purposes: they serve as a reminder for the patient of their appointment and they convey the message that they are valued and appreciated, making you feel valued in some way. It’s also worth noting that patients can make changes to their appointment or adjust their attendance, both of which cannot be done via text message." (P12)

These suggestions highlighted the need for a tailored approach to appointment reminders, utilizing both digital and personal communication methods to enhance patient engagement and attendance.

Departmental Enhancements for Improved Appointment Attendance

Participants believed that improvements within the department could significantly improve appointment attendance. These proposed improvements encompassed various aspects from logistical changes to policy reformation and informational support.

The idea of a dedicated call center for managing appointments was mentioned, with a participant suggesting,

"I think it would be better if you had a system or a call centre for the appointments, 24 hours a day, which would help us, meaning a number to assist us so that we can rest at ease." (P9)

This suggestion indicated the need for more accessible and responsive communication channels between the department and patients. The importance of a clear attendance policy was also mentioned:

"There must be a policy within the department or the hospital to manage this issue (missing appointments). In other words, when the patient misses one or two appointments, it must be rescheduled without any action taken. If the patient misses three or four appointments, stricter action may be taken." (P4)

Participants desired more flexible scheduling options to accommodate their personal and professional commitments:

"It is tough for me to attend the appointment while I am at work in the morning, and I am unable to go since we are understaffed. Perhaps you might extend the working hours till the evening or even on weekends so that I can attend my appointments." (P10)

The need for better pre-examination information in clear language was further highlighted:

"I had experienced this issue before when I was reading the preparation instruction paper and had a specific question about a certain point but did not know how to contact you. So, I had to go online and search for the information I wanted to inquire about, and as you may well know, medical information is sensitive, and you cannot trust every source you find." (P11)

 Finally, the role of marketing and informational materials in building patient trust and confidence was noted:

"You have no pamphlets in the waiting area about the method and duration of the examination or a T.V. screen that shows the hospital doctor, equipment, and success stories about diagnoses and other things. You have to show these things to encourage patients and increase their confidence, and you could have some incentives to encourage patients to attend. In addition, I did not find any advertisements for the hospital, for marketing plays a vital role in building confidence for any hospital." (P16)

This suggestion stresses the potential impact of effective communication and marketing strategies in enhancing patient engagement and trust in healthcare services. These participant recommendations further emphasized the need for departmental improvements that are patient-centric, addressing both practical and perceptual barriers to appointment attendance. Implementing these changes could lead to more efficient appointment management, better patient satisfaction, and ultimately, improved healthcare outcomes.

## Discussion

In this pioneering study conducted at the Specialist Hospital in Saudi Arabia, the reasons behind patient non-attendance at radiology appointments were explored, contributing to a deeper understanding of this issue. The study’s focus was on gathering participants’ perspectives on their non-attendance experiences and seeking their suggestions for possible solutions to mitigate the issue. This approach is crucial for enhancing the services of the radiology department, thereby improving patient health outcomes, ensuring continuity of care, expediting treatments, and managing the cost of healthcare effectively. The complex nature of the reasons influencing treatment-seeking behaviors, as observed in another study, highlights the complex nature of the issue, encompassing socioeconomic status, disease knowledge, familial and social influences, and service accessibility [24]. The findings from this study indicated that key barriers to attending appointments in the radiology department include forgetting the appointment, long waiting times, absence of reminders, difficulties in managing appointments, and inconvenient scheduling. These findings resonate with existing literature. Ngwenya and Van (2009) identified forgetfulness as the primary cause of non-attendance among diabetic patients in South Africa, and a study on outpatient appointments in the Sultanate of Oman (Royal Hospital outpatient appointments) reported similar reasons [11,25] Patients often forgot their appointments because they were scheduled too far in advance, exacerbating the challenge of keeping track of the appointment date and time. Long waiting times, a known cause of reduced appointment attendance and patient dissatisfaction, emerged as a significant issue in this study [8,17]. The specific issue of extended waiting times for MRI services can be attributed to the lengthy duration of MRI tests and the limited number of daily appointments available, compared to other tests.

Another critical reason was the appointment time. In line with previous studies, our study suggested that appointment times often clashed with patients’ personal and professional commitments, leading to non-attendance [3,16,26]. The selection of the first available time slot, which might not be ideal for the patient, increases the likelihood of non-attendance. An advanced access system allowing patients to choose suitable times may be a viable solution to address this ongoing challenge. This study recommends complex strategies to reduce non-attendance by considering the varied and complex reasons behind it.

In this study, patients expressed dissatisfaction with the quality of the interaction, and explanations, and perceived a lack of trust in their doctors. This sentiment was echoed by patients who felt that physicians did not provide sufficient information or persuasive explanations for their illnesses or the need for referrals. This finding aligned with Yang et al.’s (2020) findings, where patients perceived a lack of interaction and trust with their doctors, leading to doubts about the doctors’ motivations [18]. The root of this issue might stem from the fact that doctors, burdened with heavy patient loads, can struggle to allocate ample time for individual interactions, thereby hindering the development of meaningful patient-doctor connections. Therefore, encouraging stronger doctor-patient relationships is crucial, as building trust and rapport can significantly mitigate these negative perceptions. Additionally, particularly for female patients, challenges in attending appointments were compounded by factors like residing far from the hospital, the lack of personal transportation, cultural inhibitions about using taxis, and social constraints. This study highlighted the profound impact of geographical distance, the unavailability of a car, inadequate public transport, and the costs of travel on appointment attendance, especially for women in Saudi Arabia, a finding corroborated by studies like those of Alhamad (2013) and AlRowaili et al. (2016) [13,27].

Additionally, a lack of disease awareness was identified as a reason for non-attendance in this study. This echoed the findings from a Malaysian study where patients’ failure to recognize symptoms led to delays in seeking medical advice. Intriguingly, some participants in this study displayed a reluctance to seek medical care unless their health issues directly impacted their day-to-day lives, often adopting an unwarrantedly optimistic view of their health conditions. Therefore, improving patients’ health literacy is essential. Addressing issues in understanding and communicating medical advice can be enhanced by increasing cultural awareness among medical staff.

The findings of this study at the Specialist Hospital in Saudi Arabia also highlighted significant concerns regarding the emotional responses of patients towards attending appointments in the radiology department. Fear, anxiety, and hesitancy emerged as potent emotional barriers, potentially contributing to higher rates of non-attendance in the department. This is in line with a UK study by Eades and Alexander (2019), where patients expressed fear prior to appointments, particularly their apprehension about receiving unfavorable news from physicians [3]. Addressing these fears requires interventions that are sensitively tailored to everyone’s concerns.

Another critical aspect underlined by this study was the importance of a timely confirmation system before appointments. Over 90% of the patients who missed appointments indicated a preference for such a system. Supporting this finding, a systematic review by McLean et al. (2016) demonstrated the effectiveness of all types of reminders in reducing non-attendance. The choice of a reminder system should be based on both the service provided and the demographic characteristics of the patient population [28]. For instance, Roberts et al. (2011) found that in a younger patient group (mean age 31), text messaging was the preferred reminder method [29]. However, in this study, older patients favored telephone reminders, whereas younger patients showed a preference for text message reminders. Furthermore, patients suggested increasing the number of available appointment slots, aligning with previous research conducted in a radiology unit at a Tertiary Care Veterans Affairs Medical Centre [30].

Despite the valuable insights gleaned from this study, it is important to acknowledge its limitations as well. It focused on patient perspectives, excluding insights from medical professionals who might have provided additional vital information. Another limitation was its inability to explore the reasons contributing to patients’ attendance, as understanding these reasons could potentially determine the best practices for enhancing appointment attendance. Therefore, future research is warranted to investigate not only the barriers but also the facilitators to patient attendance at radiology appointments, thus providing a more comprehensive understanding of the reasons influencing patient behavior in clinical settings.

## Conclusions

In exploring the reasons for non-attendance, this study explored themes such as scheduling problems, inadequate knowledge about health conditions, physician-patient miscommunication, and travel difficulties. Each of these themes encompasses a range of issues. For instance, scheduling problems not only involve conflicts with personal or work commitments but also systemic issues such as long waiting times and hospital appointment management inefficiencies. Similarly, inadequate knowledge about health conditions revealed a lack of patient awareness about the importance of radiology appointments and misunderstandings about their health status. Moreover, the study explored the impact of physician-patient miscommunication, highlighting issues such as the quality of interaction, the adequacy of explanations provided by healthcare professionals, and the level of trust in the physician-patient relationship. Travel difficulties, especially significant in Saudi Arabia, include challenges related to geographical distances, the lack of personal transportation, and cultural factors affecting mobility, particularly for female patients. The insights gained from this qualitative study are invaluable for developing targeted strategies to reduce non-attendance rates. These strategies can range from improving communication and patient education to addressing logistical barriers and enhancing appointment scheduling systems. The findings also have implications for policy and practice, suggesting the need for a more patient-centric approach in healthcare service design and delivery. This study aimed to contribute to the growing body of knowledge on patient non-attendance, particularly in the context of radiology services in Saudi Arabia. By providing a detailed analysis of patient perspectives, it offers a unique lens through which the complexities of healthcare use can be understood and addressed.
